# Treatment of blood blister-like aneurysms of the supraclinoid internal carotid artery using pipeline and lattice flow diverters and coiling

**DOI:** 10.3389/fneur.2025.1607683

**Published:** 2025-07-25

**Authors:** Xuan Chen, Zibo Zhou, Jinguang Gao, Jinlu Yu

**Affiliations:** ^1^Department of Neurosurgery, The First Hospital of Jilin University, Changchun, China; ^2^Department of Neurosurgery, Yushu People’s Hospital, Changchun, China

**Keywords:** blood blister-like aneurysm, supraclinoid internal carotid artery, flow diverter, coiling, vasospasm

## Abstract

**Background:**

Flow diverters (FDs) have shown the potential to treat blood blister-like aneurysms (BBAs) of the supraclinoid internal carotid artery (ICA). We report a series of cases treated by both deploying FDs and coiling.

**Methods:**

Based on the Bojanowski classification, the BBAs of the supraclinoid ICA were classified into types I–IV. Based on the approach used for endovascular treatment (EVT) of the BBAs, the EVTs were categorized into types 1–3. The modified Rankin scale (mRS) was used to assess the clinical follow-up outcome.

**Results:**

Thirteen patients with 13 BBAs of the supraclinoid ICA who were aged 22–66 (49.8 ± 13.5) years were included. There were 12 females and 1 male. All patients had experienced subarachnoid hemorrhage. According to the Bojanowski’s et al. classification, a total of 2, 7, 3, and 1 BBAs were categorized as types I, II, III, and IV, respectively. Preoperative vasospasms were detected in 4 patients. EVT types 1, 2, and 3 were used to treat 4, 7, and 2 BBAs, respectively. After EVT, 2 patients with preoperative vasospasm experienced hemiparesis but later recovered. One patient with a preoperative vasospasm experienced multiple infarctions and died. One patient who discontinued antiplatelet therapy experienced multiple infarctions and suffered severe disability. ‌Twelve patients completed a post-EVT six-month follow-up, excluding the one with postoperative mortality.‌ The mRS scores were 0, 1, and 4 for 10, 1, and 1 patients, respectively. Eleven patients were subjected to angiographic follow-up. All BBAs were cured, and the supraclinoid ICAs were repaired.

**Conclusion:**

For patients with BBAs, deploying an FD and coiling can yield good outcomes. However, ischemic complications should not be overlooked.

## 1 Introduction

Blood blister-like aneurysms (BBAs) are rare ruptured lesions; they are often located at the supraclinoid internal carotid artery (ICA), and they account for 0.9–6.5% of all ICA aneurysms (1). BBAs of the supraclinoid ICA are located at the nonbranching site of the dorsal ICA wall; they present with a slight bulge and a conical, hemispherical, or saccular shape. These aneurysms have a focal wall defect with an absence of internal elastic lamina and media, and the arterial gap is only covered with adventitia and thin fibrinous tissue after bleeding. BBAs of the supraclinoid ICA are typically responsible for subarachnoid hemorrhage (SAH) in the absence of other adjacent associated intracranial aneurysms (2).

Due to the weak consistency of the wall, BBAs can rapidly grow. Therefore, BBAs of the supraclinoid ICA have a high risk of rerupture, which is associated with poor prognoses. Prompt treatment is necessary in these cases. Management is challenging, given the fragile walls of BBAs of the supraclinoid ICA. Previously, many surgical techniques, such as wrapping or trapping with bypass, have been described for the treatment of these lesions (3). However, these techniques are associated with high perioperative morbidity and mortality rates. Recently, endovascular treatment (EVT) has emerged as a feasible option (4).

Among EVT techniques, the flow diverter (FD) with a high metal coverage rate has shown the potential to cure BBAs of the supraclinoid ICA (5). However, several issues remain, such as the necessity of coiling assistance, BBA classification for EVT, and EVT types. Therefore, we conducted the current study to address these issues. At our institute, the Pipeline embolic device (PED) Flex (Medtronic, Irvine, CA, United States) and Lattice FD device (AccuMedical, Beijing, China) are used to treat these BBAs. Therefore, we also discussed these two devices in the context of EVT for BBAs of the supraclinoid ICA.

## 2 Materials and methods

The present study was approved by the Ethics Committee of our institute, and informed consent was obtained from the participants (No. 2025-200). Patient data were continuously collected from December 2022 to February 2025.

### 2.1 Inclusion and exclusion criteria

The inclusion criteria were as follows: (a) SAH; (b) angiography revealed a lesion on the supraclinoid ICA in accordance with the BBA diagnostic criteria (described in the Introduction section); (c) BBAs treated with both FDs and coiling; and (d) complete clinical data before treatment, after treatment, and during follow-up. Patients with BBAs treated by microsurgery or EVT without FD deployment were excluded.

### 2.2 BBA classification

This study used Bojanowski’s et al. (6) classification for BBAs. Type I BBAs have a small bulge and no neck. Type II BBAs resemble berry aneurysms and involve part of the ICA wall, but they have a sac with a large neck (although it is no larger than the diameter of the ICA). Type III BBAs involve a significantly larger longitudinal portion of the ICA, defined as longer than the diameter of the ICA. Type IV BBAs involve almost all or the entire circumference of the ICA, resulting in enlargement of the ICA on angiography.

### 2.3 Antiplatelet/anticoagulation management

At least 3 h before EVT, a loading dose of dual antiplatelet agents (aspirin 300 mg and ticagrelor 180 mg) was administered. During FD deployment and coiling, tirofiban (4–6 mL by intravenous bolus) was administered. ‌Post-EVT, intravenous tirofiban infusion was maintained at 4–6 mL/h until 2 h after the second dose of oral ticagrelor 90 mg. From the second day of EVT, antiplatelet agents (aspirin 100 mg/qd and ticagrelor 60 mg/bid) were continued until 6 months after EVT. One month after EVT, ticagrelor 60 mg/bid could be switched to clopidogrel 75 mg/qd.‌ After angiographic follow-up, ticagrelor or clopidogrel could be discontinued, but aspirin treatment continued for 6 months to 1 year or for lifetime.

### 2.4 EVT strategy

**Figure 1:**
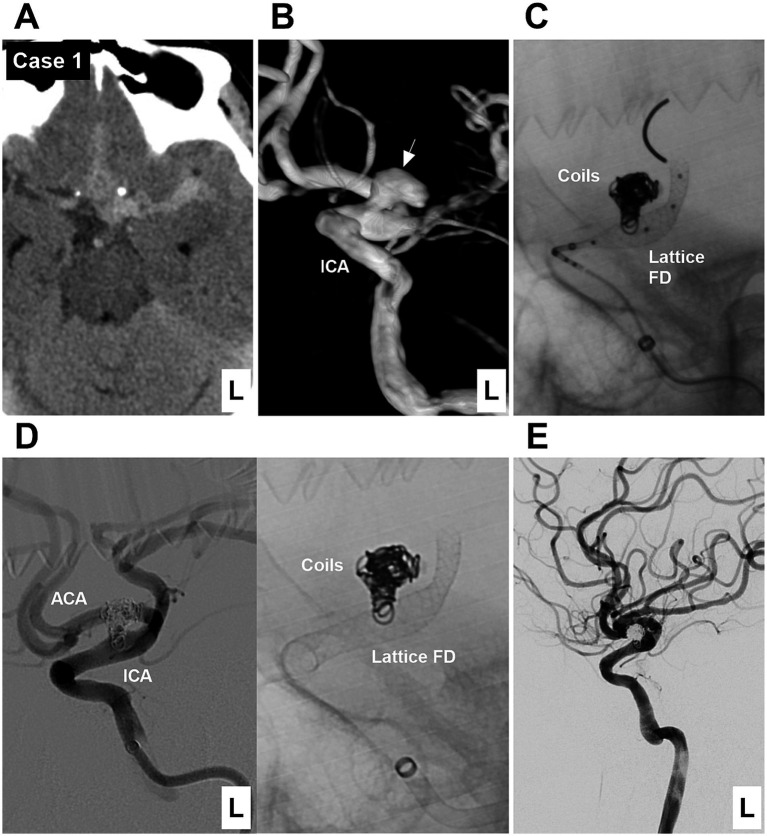
Case 1: The patient was first treated by coiling and then by deploying a Lattice FD device (Type 1 EVT). **(A)** Computed tomography showing subarachnoid hemorrhage focused on the left suprasellar cistern. **(B)** DSA showing a Type II BBA (arrow) of the left supraclinoid ICA. **(C)** X-ray image showing that the Lattice FD device covered the BBA neck after coiling the BBA. **(D)** Left panel: Postoperative DSA image showing the patency of the supraclinoid vessels. Right panel: X-ray image showing the coils and Lattice FD device. **(E)** Six-month follow-up DSA showing that the BBA was cured, and the supraclinoid ICA was repaired. ACA, anterior cerebral artery; BBA, blood blister-like aneurysm; DSA, digital subtraction angiography; EVT, endovascular treatment; FD, flow diverter; ICA, internal carotid artery; L, left.

**Figure 2:**
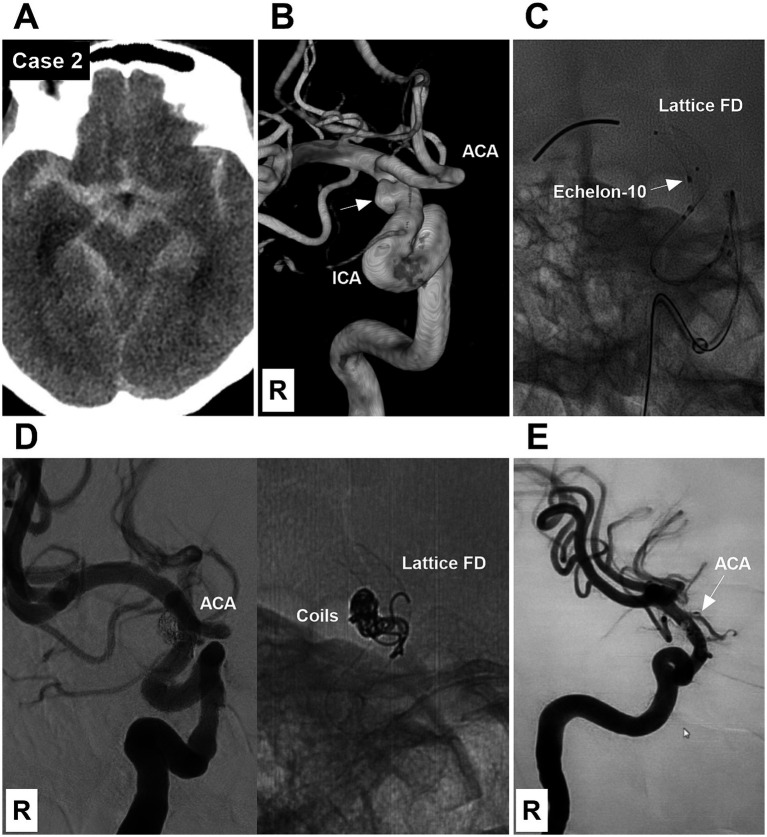
Case 2: The patient was treated by both coiling and deploying a Lattice FD device (Type 2 EVT). **(A)** Computed tomography showing diffuse subarachnoid hemorrhage of the suprasellar cistern. **(B)** DSA showing a Type II BBA (arrow) of the right supraclinoid ICA. **(C)** X-ray image showing an Echelon-10 microcatheter tip (arrow) at the BBA neck and a partially deployed Lattice FD device beyond the BBA neck. **(D)** Left panel: Postoperative DSA showing the BBA embolized. Right panel: X-ray image showing the coils and Lattice FD device. **(E)** Six-month follow-up DSA showing that the BBA was cured and the supraclinoid ICA was repaired. However, the right ACA (arrow), covered by the FD, was nearly occluded. ACA, anterior cerebral artery; BBA, blood blister-like aneurysm; DSA, digital subtraction angiography; EVT, endovascular treatment; FD, flow diverter; ICA, internal carotid artery; R, right.

**Figure 3:**
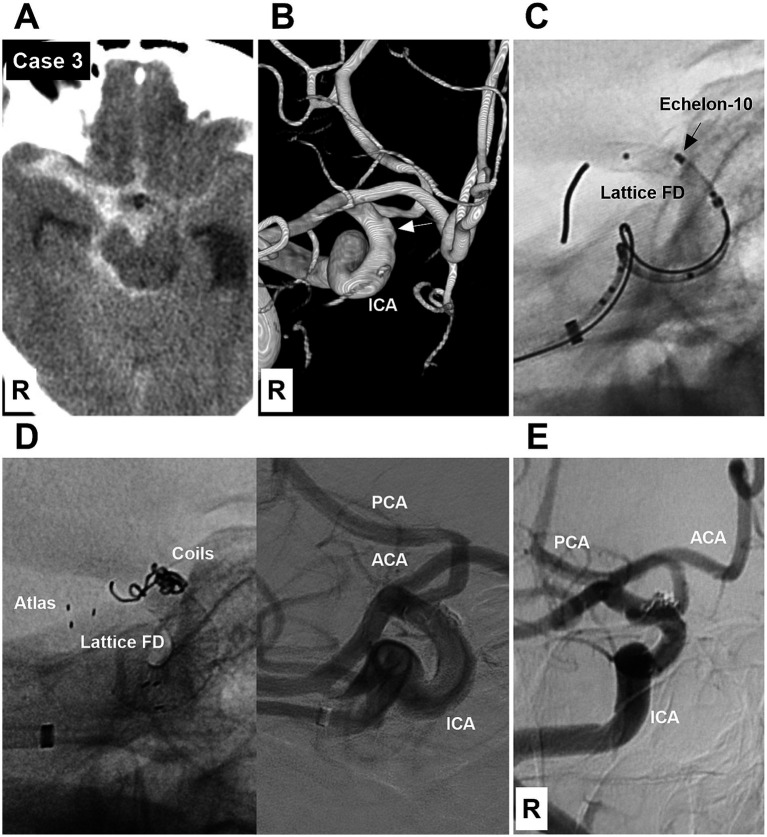
Case 3: The patient was treated by both coiling and deploying a Lattice FD device and a Neuroform Atlas stent (Type 2 EVT). **(A)** Computed tomography showing subarachnoid hemorrhage focused on the right suprasellar cistern. **(B)** DSA showing a Type I BBA (arrow) of the right supraclinoid ICA. **(C)** X-ray image showing an Echelon-10 microcatheter tip (arrow) at the BBA neck and a partially deployed Lattice FD device beyond the BBA neck. **(D)** Left panel: X-ray image showing that the BBA was embolized, and the Neuroform Atlas stent was used to fix the coil loop that escaped from the BBA due to the foreshortening of the Lattice FD device after deployment. Right panel: Postoperative DSA showing the patency of the supraclinoid vessels. **(E)** Six-month follow-up DSA showing that the BBA was cured, and the supraclinoid ICA was repaired. ACA, anterior cerebral artery; BBA, blood blister-like aneurysm; DSA, digital subtraction angiography; EVT, endovascular treatment; FD, flow diverter; ICA, internal carotid artery; PCA, posterior cerebral artery; R, right.

**Figure 4:**
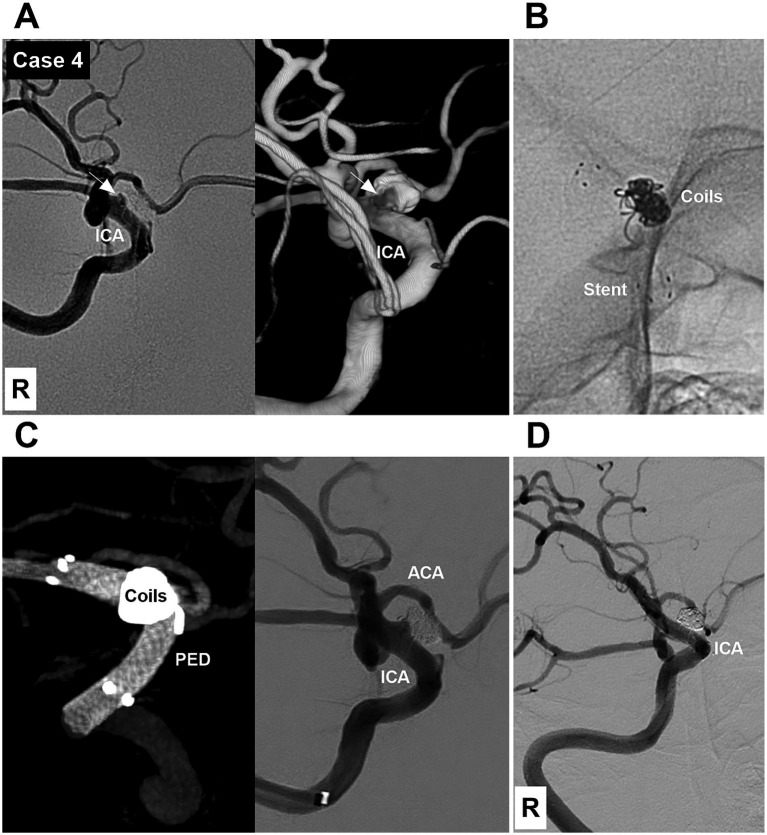
Case 4: The patient was treated by deploying a PED after incomplete EVT (Type 3 EVT). **(A)** DSA (left panel) and reconstructive DSA (right panel) images showing a Type II BBA with a residual neck (arrows) of the right supraclinoid ICA after incomplete EVT. **(B)** X-ray image showing the coils and a low-metal-coverage stent. **(C)** Left panel: Vaso-DSA showing that the PED was deployed to cover the BBA in the low-metal coverage stent. Right panel: Postoperative DSA showing the patency of the right supraclinoid vessels. **(D)** Six-month follow-up DSA showing that the BBA was cured and the supraclinoid ICA was repaired. ACA, anterior cerebral artery; BBA, blood blister-like aneurysm; DSA, digital subtraction angiography; EVT, endovascular treatment; ICA, internal carotid artery; PED, Pipeline embolic device; R, right.

EVT was performed by a transfemoral approach under general anesthesia. A combination of a long sheath and a distal access catheter was used to provide sufficient support. The distal access catheter reached the cavernous segment of the ICA. BBAs were subsequently treated via FDs and coiling. EVT can be divided into three types according to the method of deploying an FD and coiling the BBA. Type 1 EVT involves first coiling the BBA sac and then releasing the FD to cover the BBA (Figure 1). Type 2 EVT involves releasing a partial FD to help coil the BBA and then completely releasing the FD to cover the BBA (Figures 2, 3). Type 3 EVT involves only deploying the FD to cover the recurrent BBA (Figure 4).

### 2.5 Recorded data

The following data were recorded: patient age and sex, presentation, Hunt–Hess grade of SAH, BBA Bojanowski classification, BBA size and location, preoperative vasospasm of the ICA and middle cerebral artery (MCA), EVT type, post-EVT event, follow-up aneurysm occlusion and ICA status, and modified Rankin scale (mRS) score.

## 3 Results

### 3.1 General information

A total of 13 patients with 13 BBAs that affected the supraclinoid ICA, who were aged 22–66 (49.8 ± 13.5) years, were included.‌ There were 12 females and 1 male. Ten patients were admitted for SAH, and 1 patient had intracerebral and intraventricular hemorrhages; among these 11 patients, the Hunt–Hess grades were I, II, and III in 3, 6, and 2 patients, respectively. Two patients who experienced previous SAH underwent incomplete EVT. Upon admission, these 2 patients were asymptomatic, and the Hunt–Hess grade was recorded as 0.

### 3.2 Angiographic classification and EVT type

According to Bojanowski’s et al. classification, type I, II, III, and IV BBAs were detected in 2, 7, 3, and 1 patients, respectively. The size of the BBAs ranged from 2–5 mm (3.8 ± 1.1). The BBAs were located in the left and right supraclinoid ICAs in 5 and 8 patients, respectively. Preoperative vasospasm of the ICA and MCA was found in 4 patients. EVT types 1, 2, and 3 were used to treat 4, 7, and 2 BBAs, respectively. Among the 13 cases in which EVT was administered, 8 PED devices and 5 Lattice FDs were used. One EVT involved a Neuroform Atlas stent (Stryker Neurovascular, Fremont, CA, United States) (Case 3; Figure 3).

### 3.3 EVT outcomes and follow-up

**Figure 5:**
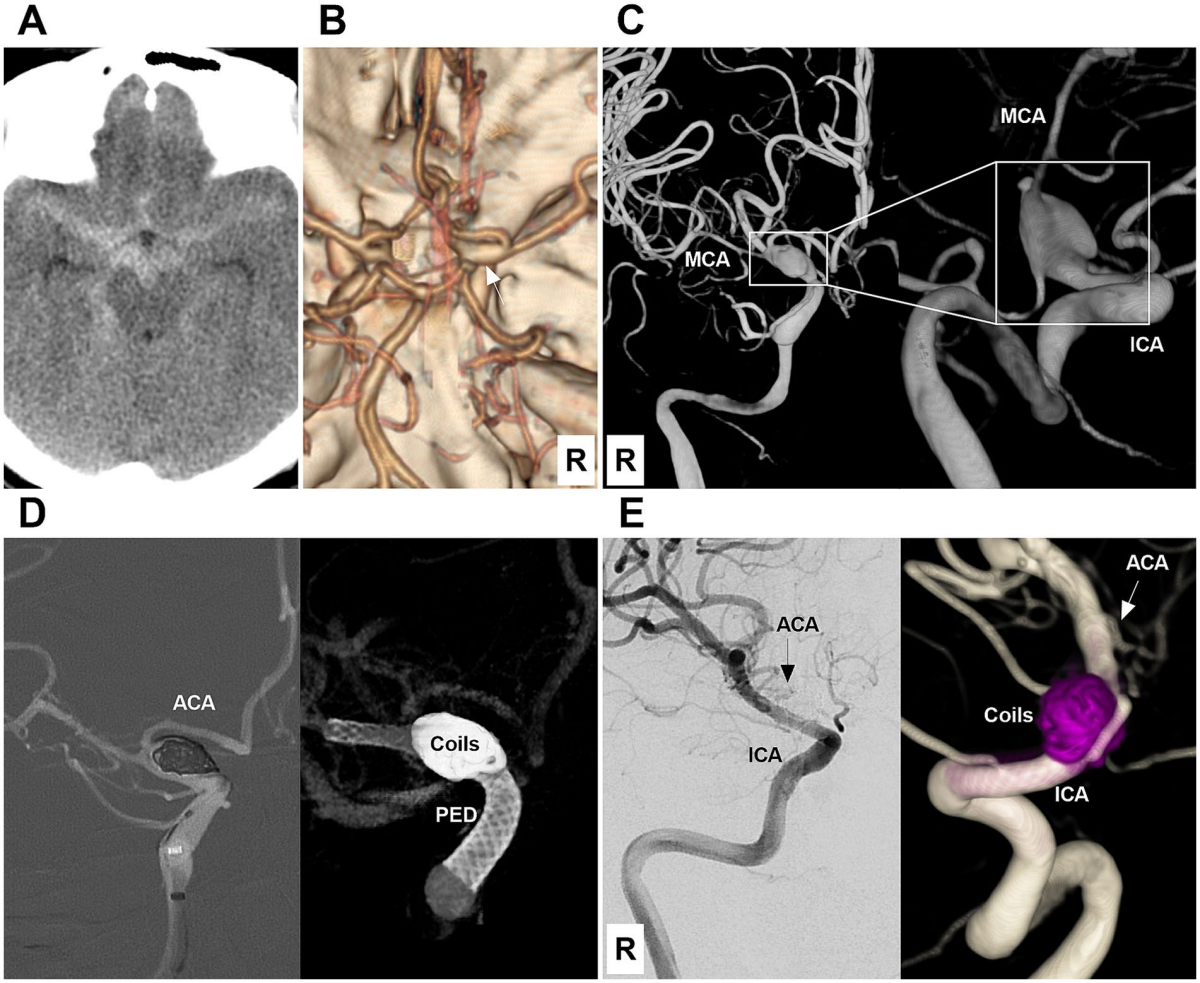
Case 5: The patient was first treated by coiling and then by deployment of a PED (Type 1 EVT). **(A)** Computed tomography showing diffuse subarachnoid hemorrhage of the suprasellar cistern. **(B)** Computed tomography angiography showing mild dilatation (arrow) of the right intracranial ICA terminus. **(C)** DSA images showing a Type IV BBA (frames) of the right supraclinoid ICA. The right MCA was thin due to vasospasm. **(D)** Left panel: Roadmap image showing that the BBA was coiled first. Right panel: Vaso-DSA showing that the PED was deployed to cover the BBA neck. **(E)** Six-month follow-up DSA (left panel) and reconstructive (right panel) images showing that the BBA was cured and the supraclinoid ICA was repaired. The right ACA (arrows) was nearly occluded by PED coverage. ACA, anterior cerebral artery; BBA, blood blister-like aneurysm; DSA, digital subtraction angiography; ICA, internal carotid artery; MCA, middle cerebral artery; PED, Pipeline embolic device; R, right.

**Figure 6:**
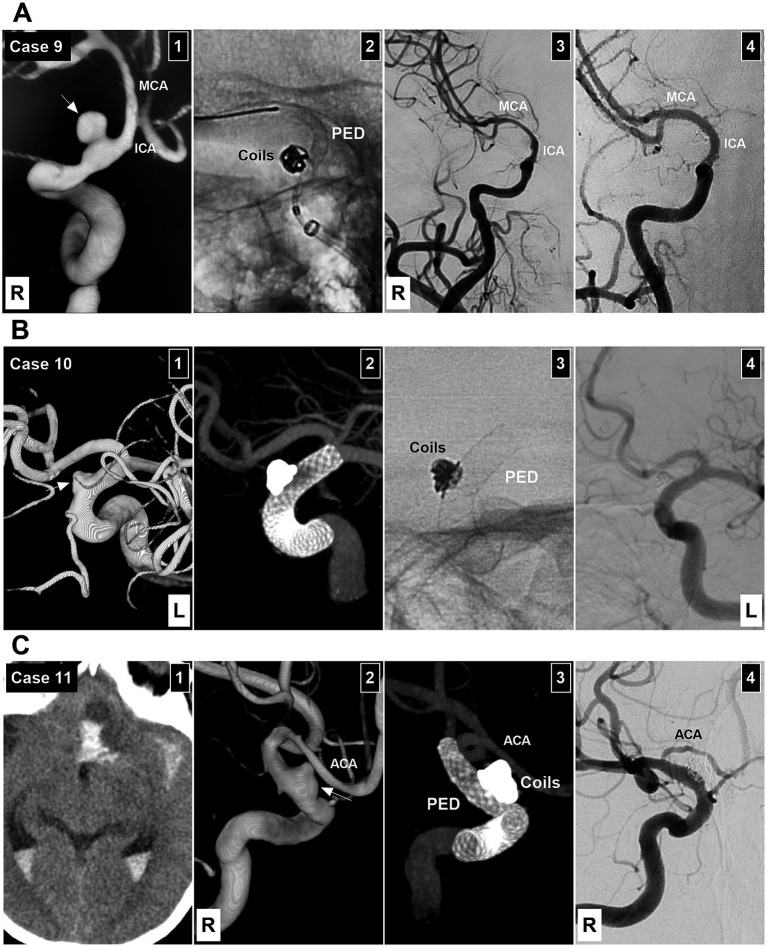
EVTs of Patients 9–11. **(A)** Patient 9 treated with Type 1 EVT. Panel 1: DSA showing a Type II BBA (arrow) of the right supraclinoid ICA, the ICA, and MCA were thin due to vasospasm. Panel 2: X-ray image showing the PED partially deployed after coiling the BBA. Panel 3: Postoperative DSA showing the embolized BBA. Panel 4: Six-month follow-up DSA showing complete aneurysm occlusion and supraclinoid ICA repair, with normal ICA and MCA size. **(B)** Patient 10 treated with Type 2 EVT. Panel 1: DSA showing a left Type II BBA (arrow). Panel 2: Vaso-DSA showing the patency of the supraclinoid vessels. Panel 3: X-ray image showing the coils and PED. Panel 4: Six-month follow-up DSA showing complete aneurysm occlusion and supraclinoid ICA repair. **(C)** Patient 11 treated with Type 2 EVT. Panel 1: Computed tomography showing multiple subarachnoid, intraventricular, and parenchymal hemorrhages. Panel 2: DSA showing a right Type II BBA (arrow). Panel 3: Vaso-DSA showing the BBA coiled by the combination of deploying FD. Panel 4: Six-month follow-up DSA showing complete aneurysm occlusion and supraclinoid ICA repair; the right ACA was stenotic after PED coverage. ACA, anterior cerebral artery; BBA, blood blister-like aneurysm; DSA, digital subtraction angiography; EVT, endovascular treatment; ICA, internal carotid artery; L, left; MCA, middle cerebral artery; PED, Pipeline embolic device; R, right.

**Figure 7:**
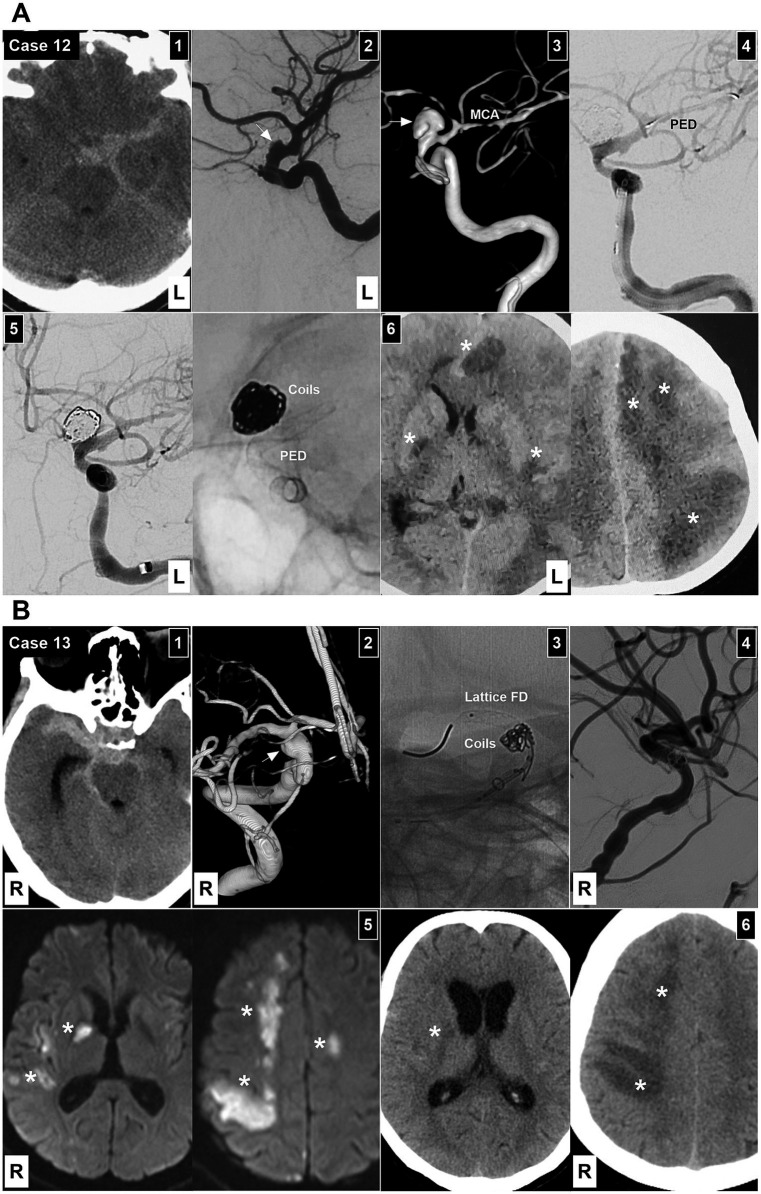
EVT in Patients 12 and 13. **(A)** Patient 12 treated by Type 1 EVT. Panel 1: Computed tomography showing subarachnoid hemorrhage focusing on the left suprasellar cistern. Panel 2: At the time of onset, DSA showing a Type III BBA of the left supraclinoid ICA (arrow). Panel 3: After 1 week, DSA showing the enlargement of the BBA (arrow). Panel 4: DSA showing the PED across the BBA neck after coiling the BBA. Panel 5: Left panel: DSA showing the BBA embolized. Right panel: X-ray image showing the coils and PED. Panel 6: One-week postoperative computed tomography showing diffuse multiple infarctions (asterisks) of the bilateral hemispheres. **(B)** Patient 13 treated with Type 2 EVT. Panel 1: Computed tomography showing subarachnoid hemorrhage focusing on the right suprasellar cistern. Panel 2: DSA image showing a Type III BBA of the right supraclinoid ICA (arrow). Panel 3: X-ray image showing the Lattice FD partially deployed to assist in coiling the BBA. Panel 4: DSA showing the embolized BBA. Panel 5: After 1 week, diffusion-weighted magnetic resonance image showing multiple infarctions (asterisks). Panel 6: After 2 weeks, computed tomography showing multiple infarctions (asterisks). BBA, blood blister-like aneurysm; DSA, digital subtraction angiography; EVT, endovascular treatment; FD, flow diverter; ICA, internal carotid artery; L, left; MCA, middle cerebral artery; PED, Pipeline embolic device; R, right.

EVT was successful in all patients (Figures 1–7). There were no procedure-related complications. After EVT, two patients (Case 5 and Case 9) with preoperative vasospasms experienced hemiparesis. These two patients subsequently recovered. After EVT, one patient (Case 12) with a preoperative vasospasm developed multiple infarctions of the bilateral hemispheres and died. One patient (Case 13) discontinued dual antiplatelet therapy and subsequently developed multiple infarctions of the bilateral hemispheres, resulting in severe disability. Of the 13 patients, 12 underwent a clinical follow-up for 6 months after EVT, excluding the one with postoperative mortality.‌ The mRS scores were 0, 1, and 4 for 10, 1 and 1 patients, respectively. Among the 12 surviving patients, one patient (Case 13), who refused the angiographic follow-up, had severe disability, and 11 patients were subjected to angiographic follow-up for 6 months. During follow-up digital subtraction angiography, all BBAs were cured, and the supraclinoid ICAs were repaired. The detailed clinical data are shown in Table 1.

**Table 1:** Clinical data of the patients.

Case	Age/Sex	Presentation	HH grade	Bojanowski classification	Aneurysm size	Side	ICA and MCA vasospasm	EVT	Post-EVT event	Aneurysm occlusion/ICA size at 6-month follow-up	mRS
1	59/M	SAH	I	II	5 mm	L	No	Type 1: Lattice FD + Coiling	No	Complete/Normal size	0
2	22/F	SAH	II	II	4 mm	R	No	Type 2: Lattice FD + Coiling	No	Complete/Normal size	0
3	48/F	SAH	II	I	2 mm	R	No	Type 2: Lattice FD + Atlas + Coiling	No	Complete/Normal size	0
4	63/F	SAH/Incomplete EVT	0	II	5 mm	R	No	Type 3: PED	No	Complete/Normal size	0
5	33/F	SAH	I	IV	5 mm	R	Yes	Type 1: PED + Coiling	Hemiparesis	Complete/Normal size	0
6	35/F	SAH	II	II	4 mm	L	No	Type 2: Lattice FD + Coiling	No	Complete/Normal size	0
7	46/F	SAH	II	I	2 mm	R	Yes	Type 2: PED + Coiling	No	Complete/Normal size	0
8	52/F	SAH/Incomplete EVT	0	II	4 mm	L	No	Type 3: PED	No	Complete/Normal size	0
9	61/F	SAH	III	II	4 mm	R	Yes	Type 1: PED + Coiling	Hemiparesis	Complete/Normal size	0
10	66/F	SAH	I	II	3 mm	L	No	Type 2: PED + Coiling	No	Complete/Normal size	0
11	65/F	SAH + IH + IVH	III	III	4 mm	R	No	Type 2: PED + Coiling	No	Complete/Normal size	1
12	50/F	SAH	II	III	5 mm	L	Yes	Type 1: PED + Coiling	Multiple infarctions	No	Deceased
13	47/F	SAH	II	III	3 mm	R	No	Type 2: Lattice FD + Coiling	Multiple infarctions	No	4

EVT, endovascular treatment; F, female; FD, flow diverter; HH, Hunt Hess; ICA, internal carotid artery; IH, intracerebral hemorrhage; IVH, intraventricular hemorrhage; L, left; M, male; MCA, middle cerebral artery; mRS, modified Rankin scale; PED, Pipeline embolic device; R, right; SAH, subarachnoid hemorrhage.

## 4 Discussion

BBAs predominantly occur in middle-aged females and are commonly located in the ICA (7). In our report, 12 of 13 patients were female, averaging 49.8 years, aligning with the above characteristics. BBAs only consist of a platelet plug covering a thin l adventitia, with a defect in the intima and media that lacks the usual collagen layer belonging to pseudoaneurysms. Therefore, management remains challenging (2). To date, no consensus has been reached regarding the optimal treatment for BBAs of the supraclinoid ICA. Current therapies include aneurysm clipping or wrapping, endovascular or surgical trapping with or without extra bypass, coiling, and deploying multiple overlapping stents, covered stents, and FDs et al. (4, 8).

Among all the treatment options for BBAs of the supraclinoid ICA, FDs are an attractive option (5). With a > 30% metal coverage rate, FDs can reconstruct the supraclinoid ICA, redirect blood flow, and occlude the BBA, providing sufficient protection for these lesions. FDs can be used to cure BBAs. In 2025, Jin et al. (7) conducted a systematic review and meta-analysis of 30 studies involving 311 BBAs treated by FDs. They found that 76.3% of BBAs were in the ICA, with 85% of BBAs achieving complete occlusion after EVT. Furthermore, 84% of patients experienced an mRS score of 0–2, and the overall and periprocedural complications account for 16.8 and 9.1%, respectively. In 2023, Zhang et al. (1) conducted a pooled analysis of 233 ICA-BBAs treated by FDs. The outcomes revealed a complete occlusion rate of 79%, a recurrence rate of 2%, a perioperative stroke rate of 8%, a perioperative mortality rate of 4%, a long-term good clinical outcome rate of 85%, and a mortality rate of 6%.

In our report, complete aneurysm occlusion was achieved in 11 BBAs, as confirmed by a 6-month angiographic follow-up. This study used two types of FDs, including 8 PEDs and 5 Lattice FDs. PED Flex is a braided tube consisting of 48 strands with 75% cobalt chromium and 25% platinum. It can provide a 30% metal coverage when deployed within a matched vessel (9). The Lattice FD device is a Chinese product with compressed mechanical balloons. The mechanical balloons will expand, assisting during the device opening. Due to mechanical balloon incorporation in the design, when the device is deployed, the forward movement of the distal wire is minimal. The Lattice FD device comprises 36 cobalt-chromium wires and 12 platinum-tungsten wires, providing a metal coverage rate of 30–40% (10).

Although FD deployment to treat BBAs has shown patency, it is uncertain whether coiling is a necessary addition (11). According to Zhang’s et al. (1) analysis, the total occlusion rate of both deploying an FD and coiling is higher than that in the FD only subgroup, at 98 and 87%, respectively. The incidence rates of favorable prognostic outcomes in the FD and coiling subgroup and the FD only subgroup were 93.1 and 81.9%, respectively. Therefore, the combination of both deploying an FD and coiling is an effective treatment approach, as it can minimize the hemodynamic burden of the fragile BBA dome and accelerate thrombosis. In our study, all BBAs were treated by both deploying an FD and coiling, leading to favorable outcomes.

When performing FD deployment and coiling, BBA types were based on the EVT type. As described in the EVT strategy section, selecting the EVT approach according to BBA types was reasonable. This is the first study to propose the principle of using deploying FD and coiling in combination, which is highlighted in our report. However, for BBA EVT by deploying FD, the complication of severe vasospasm had to be considered. Preoperative vasospasms can be found in BBAs caused by SAH. In our study, 4 patients exhibited preoperative vasospasms, which resulted in multiple infarctions in 1 patient who died. However, one patient without a preoperative vasospasm suffered multiple infarctions and experienced severe disability, which was due to voluntary discontinuation of dual antiplatelet therapy. It was unclear how to avoid postoperative severe ischemic complications. However, when treating patients with preoperative vasospasms, both deploying an FD and coiling should be performed with caution. Once this type of the EVT is performed, adequate antiplatelet management and a sufficient blood volume may be necessary.

### 4.1 Limitations

This was a retrospective single-center study with a limited sample size. Only 13 patients were included, which may have led to biased results and limited generalizability of the conclusions. The EVT classification has been proposed by Bojanowski, but whether this classification can accurately guide treatment strategies and prognosis assessment requires validation in studies with larger sample sizes. In this study, the follow-up was conducted for only 6 months. For BBAs, a condition with the potential for long-term recurrence, the absence of longer-term follow-up data prevents a comprehensive assessment of long-term efficacy.

## 5 Conclusion

For BBAs of the supraclinoid ICA, deploying Pipeline and Lattice FDs and coiling can yield favorable clinical and angiographic outcomes. However, ischemic complications from vasospasm should not be overlooked.

## Data Availability

The raw data supporting the conclusions of this article will be made available by the authors, without undue reservation.
